# The TMD-7 as a Brief Measure for Assessing Temporomandibular Disorder

**DOI:** 10.1055/s-0042-1746416

**Published:** 2022-08-09

**Authors:** Emily B. Koufos, Harold C. Avila, George Eckert, Kelton T. Stewart, Kurt Kroenke, Hakan Turkkahraman

**Affiliations:** 1 Department of Orthodontics and Oral Facial Genetics, School of Dentistry, Indiana University, Indianapolis, Indiana; 2 Department of Oral Pathology, Medicine and Radiology, School of Dentistry, Indiana University, Indianapolis, Indiana; 3 Department of Biostatistics and Health Data Science, School of Medicine, Indiana University, Indianapolis, Indiana; 4 Regenstrief Institute, Indiana University, Indianapolis, Indiana

**Keywords:** headache, pain, temporomandibular disorders, orthodontic treatment

## Abstract

**Objectives** The aim of this cross-sectional prospective study was to determine the internal consistency of the TMD-7, and compare prevalence of TMD symptoms in an adult population.

**Materials and Methods** Upon presenting to the orthodontic screening appointment, a total of 440 subjects (316 females and 124 males) were asked to complete the TMD-7 questionnaire. A total of 108 of the participants were later excluded from the study either due to the duplicate or missing responses. The final sample consisted of data from 332 participants (232 females and 100 males), aged between 18 and 64 (mean age: 42.9 ± 9.0) years.

**Statistical Analysis** Cronbach's *α* statistics were calculated to assess internal consistency. Comparisons between genders, among age categories, and between subjects with versus without prior orthodontic treatment were performed using Wilcoxon ranks sum and Kruskal–Wallis tests. Comparisons for differences in the individual TMD-7 item ratings were performed using Mantel–Haenszel chi-square tests for ordered categorical responses.

**Results** The calculated Cronbach's *α* for TMD-7 scale was 0.77. No statistically significant differences were found in the TMD-7 scale score or the individual TMD-7 item ratings between age categories ( *p* = 0.993). Females had significantly higher TMD-7 scale score and higher ratings for headache, pain in jaw, pain in neck, pain in forehead, difficulty opening mouth, and difficulty while eating ( *p* < 0.05). No statistically significant differences were found in the TMD-7 scale score or the individual TMD-7 item ratings between subjects with versus without previous orthodontic treatment ( *p* = 0.075).

**Conclusion** The TMD-7 tool has good internal consistency and can be used reliably for assessment of TMD symptoms in adults. The use of this tool revealed no significant differences between age groups or between subjects with or without previous orthodontic treatment. However, a significant female gender predisposition for TMD symptoms in the adulthood was determined.

## Introduction

Temporomandibular disorder (TMD) is a medical term used to characterize multiple conditions affecting the masticatory muscles, mandibular movement, temporomandibular joint (TMJ), and surrounding structures. 1 The etiology of this multifactorial disorder results from trauma, parafunctional habits, psychological factors, occlusion, and stress resulting in a variety of symptoms ranging from muscular pain, earaches, headaches, TMJ clicking and popping, occlusal dysfunction, limited mandibular movements, and intra-articular disc pain. 1 2

Epidemiology studies have shown the prevalence of TMD ranges from 5 to 12% and with gender predilection in females. 1 3 In a survey conducted by the National Health Interview Survey between 2017 and 2018, the prevalence of TMD in the United States adult population was reported as 4.8% ranging from age 18 to 74 years of age and is the most common chronic orofacial pain disorder. 3 Chronic pain is estimated to affect 50 to 100 million U.S. adults, and other chronic comorbidities are noted to accompany TMD like fibromyalgia, chronic low back pain, and migraines. 4 When assessing specific symptoms of TMD, prevalence increases up to 50% of the adult population. 2 5 6

These disorders can result in a negative impact on daily life due to chronicity and severity of symptoms, yet these conditions are not easily detected and even neglected in dentistry and medicine. This is illustrated by a discrepancy between estimated treatment need, traceable performed treatment, and lack of evidence-based studies indicating treatment success. 3 This lack of evidence-based research in TMD contributes to underdiagnosis and inadequate treatment, despite patients seeking care from dental health professionals.

Several studies exist on assessing validity of screening tools that either addresses pain or function but have led to the conclusion of low sensitivity and high specificity of screening tools. 7 8 9 10 11 12 Gerstner et al 8 evaluated a questionnaire to distinguish patients with TMD, tension-type headaches, and controls and found the eight-question questionnaire to be reliable with high sensitivity and specificity. However, their study sample was not adequate to reach this inference. Additionally, their conclusions showed the questionnaire only distinguished reliability between the controls and TMD group and was unable to distinguish between TMD and tension-type headaches. 8

A study regarding a three-question survey by Lövgren et al 9 10 was the first study to validate a screening tool, consisting of two questions regarding pain of the joint and a third question about function. This tool was compared using the Diagnostic Criteria for TMDs. 13 The survey was deemed valid. However, a limitation of the study was the pain questions had a lower sensitivity due to how the questionnaire asked about frequency of symptoms, thus further diagnostics were needed to determine if pain in a TMD population was of TMD origin. 9 10 Due to this low sensitivity of screening tools affecting a large potential of the adult population, it is critical to provide tools for early diagnosis and provide knowledge to practitioners regarding TMD.

**Fig. 1:**
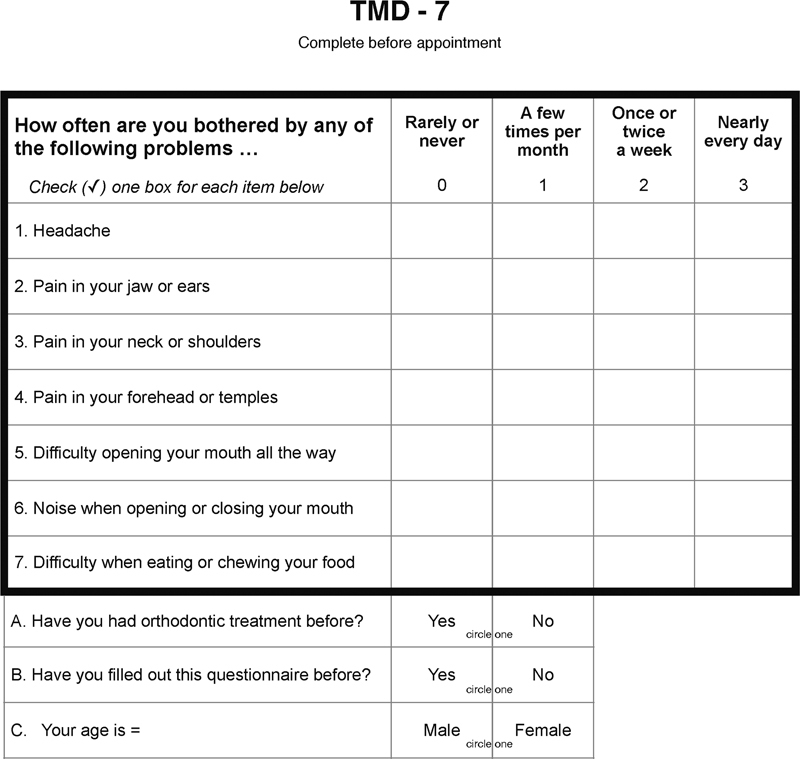
TMD-7 questionnaire.

Several features of pain and function may be identified through self-reporting by the patient. These features include symptom identification, onset, frequency, and severity. The goal for using a self-reporting instrument is to provide the health care professional quick, simple, and reliable information to help the patient make an informed decision regarding treatment(s). For these purposes, a novel measure, TMD-7, was assembled over a period beginning in November 2019 and ending in May 2020. The measure includes pain (questions 1–4) and function (questions 5–7) features ( Fig. 1 ). A window into the patient's pain can be viewed by looking at the patient's pain severity, pain frequency, pain duration, pain impact on patient's life, and pain onset. The purpose of the TMD-7 is to provide a brief measure for the patient to complete which provides enough information for a provider to confidently refer a patient for treatment. The TMD-7 records the patient's pain frequency as a glimpse into their pain complex.

This study is the first to evaluate the internal consistency of TMD-7 to assess symptoms of TMD. In addition, the study examined the prevalence of TMD symptoms in an adult population, determined whether gender predominance of TMD symptoms exists, and compared TMD symptoms in adults with or without prior orthodontic treatment.

## Materials and Methods

### Ethical Approval

The study was reviewed and approved by the Indiana University Institutional Review Board, #2009072510.

### Study Design

This cross-sectional prospective study was conducted at the Indiana University School of Dentistry, Department of Orthodontics and Oral Facial Genetics. The study population consisted of males and females who presented to a screening appointment for themselves or with a minor under their guardianship. Due to the ease of research and expedited data collection, convenience sampling method was used. A power analysis revealed that a minimum of 300 subjects was deemed necessary for this study. With a sample size of 300 subjects, the 95% confidence interval (CI) for Cronbach's *α* for the pain and function subscales would have a width of 0.12, assuming a two-sided interval and Cronbach's *α* 0.7.

### Inclusion and Exclusion Criteria

Subjects 18 years of age or older were included in the study. Eligible subjects who refused to complete the questionnaire, had already participated in the study, or were unable to read and understand the English language were excluded from the study. A written informed consent and assent were obtained from all subjects included.

### Enrollment Procedure

Upon presenting to the orthodontic screening appointment, a total of 440 subjects (316 females and 124 males) were asked to complete the TMD-7 questionnaire. Subjects recorded their responses on a paper version of the questionnaire and returned the completed questionnaire for data entry.

### Data Collection

Study data were collected and managed using REDCap electronic data capture tools hosted at Indiana University.

### TMD-7 Scoring

The participants' responses were weighted based on the increasing frequency of their pain according to the following scale: rarely or never: 0; a few times per month: 1; once or twice a week: 2; nearly every day: 3. Sum scores ranged from 0 to 21.

### Statistical Analysis

Confirmatory factor analysis (CFA) was used to evaluate whether the seven items fell into the two proposed pain and function subscale domains. Internal consistency validity was evaluated using Cronbach's *α* . Comparisons between gender, among age categories, and between subjects with and without prior orthodontic treatment for differences in the TMD-7 scale were performed using nonparametric Wilcoxon ranks sum tests and Kruskal–Wallis tests. Comparisons for differences in the individual TMD-7 item ratings were performed using Mantel–Haenszel chi-square tests for ordered categorical responses. A 5% significance level was used for all tests. Analyses were performed using SAS version 9.4 (SAS Institute, Inc., Cary, North Carolina, United States).

## Results

A total of 108 of the participants were later excluded from the study either due to duplicate or missing responses. The final sample consisted of data from 332 participants (232 females and 100 males). For age distribution, the majority of participants were over age 35 (mean 42.9 ± 9 years). The subjects were grouped into three categories for their age distribution: 18 to 35 (18%), 36 to 50 (63%), and greater than 50 years of age (19%). For orthodontic treatment, 206 (62%) subjects did not receive prior orthodontic treatment. Table 1 shows the frequency distribution for the TMD-7 individual items. The most commonly experienced symptom was pain in neck (65%) followed by headache (64%) at any rate.

**Table 1:** Frequency of responses for individual TMD-7 items

Item	Rarely or never	A few times per month	Once or twice a week	Nearly every day
Headache	121 (36%)	126 (38%)	68 (20%)	17 (5%)
Pain in jaw	224 (67%)	69 (21%)	28 (8%)	11 (3%)
Pain in neck	117 (35%)	106 (32%)	59 (18%)	50 (15%)
Pain in forehead	189 (57%)	92 (28%)	41 (12%)	10 (3%)
Difficulty when opening mouth	284 (86%)	31 (9%)	10 (3%)	7 (2%)
Noise when opening closing mouth	245 (74%)	42 (13%)	13 (4%)	32 (10%)
Difficulty while eating	283 (85%)	26 (8%)	16 (5%)	7 (2%)

### Summary Statistics

**Fig. 2:**
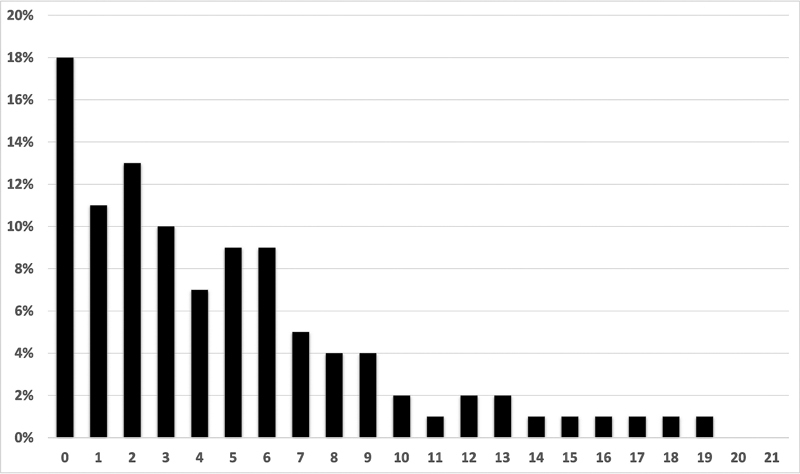
Histogram showing the distribution of the sum scores.

Table 2 shows the summary statistics which were calculated as the average and the sum of the 7 items. The mean average score for TMD-7 scale was 0.59 ± 0.54, while the mean sum score was 4.11 ± 3.76. Distributions for both average and sum scores are given in Table 3 and shown as a histogram in Fig. 2 .

**Table 2:** Summary statistics calculated as the average and the sum of the TMD-7 items

	Mean	SD	SE	Min	Max
Average score	0.59	0.54	0.03	0	2.71
Sum score	4.11	3.76	0.21	0	19

Abbreviations: SD, standard deviation; SE, standard error.

**Table 3:** Average and sum score distributions

Sum score	Average score	*N* (%) for individual score	*N* (%) cumulative
0	0	60 (18)	60 (18)
1	0.14	38 (11)	98 (30)
2	0.29	44 (13)	142 (43)
3	0.43	34 (10)	176 (53)
4	0.57	23 (7)	199 (60)
5	0.71	30 (9)	229 (69)
6	0.86	30 (9)	259 (78)
7	1	17 (5)	276 (83)
8	1.14	13 (4)	289 (87)
9	1.29	13 (4)	302 (91)
10	1.43	8 (2)	310 (93)
11	1.57	4 (1)	314 (95)
12	1.71	6 (2)	320 (96)
13	1.86	5 (2)	325 (98)
14	2	1 (< 1)	326 (98)
15	2.14	3 (1)	329 (99)
17	2.43	1 (< 1)	330 (99)
18	2.57	1 (< 1)	331 (100)
19	2.71	1 (< 1)	332 (100)
20	2.86	0 (0)	332 (100)
21	3	0 (0)	332 (100)

### Confirmatory Factor and Psychometric Analyses

The calculated Cronbach's *α* for the TMD-7 scale was 0.77, which was above the minimum acceptable value. There was a positive correlation between the seven items. Overall, all these items had a direct correlation with a range of 0.17 to 0.59 ( Table 4 ). The highest correlations seen were between headache and pain in forehead and between difficulty opening mouth and noise when opening and closing 0.59.

**Table 4:** Correlations between items of the TMD-7

Item	Headache	Pain in jaw	Pain in neck	Pain in forehead	Difficulty when opening mouth	Noise when opening closing mouth	Difficulty while eating
Headache	–						
Pain in jaw	0.32	–					
Pain in neck	0.42	0.29	–				
Pain in forehead	0.59	0.44	0.44	–			
Difficulty when opening mouth	0.21	0.41	0.27	0.17	–		
Noise when opening closing mouth	0.21	0.37	0.22	0.18	0.59	–	
Difficulty while eating	0.17	0.41	0.22	0.18	0.44	0.46	–

Initially, the TMD-7 tool was proposed as 2-factor scale: 4-item pain subscale (headache, pain in jaw, pain in neck, pain in forehead) and 3-item function subscale (difficulty opening mouth, noise opening closing, difficulty when eating). CFA failed ( *p* < 0.001, root mean square error of approximation [RMSEA] = 0.115). Thus, an exploratory factor analysis (EFA) was conducted to identify which items best paired to form subscales. The two factors identified by EFA were: a 3-item factor (headache, pain in neck, pain in forehead) and a 4-item factor (pain in jaw, difficulty when opening mouth, noise when opening closing, difficulty while eating); however, this 2-factor structure also did not satisfy CFA ( *p* < 0.001, RMSEA = 0.103) ( Table 5 ).

**Table 5:** Factor structure of TMD-7 after promax oblique rotation

Item	Factor 1 (function)	Factor 2 (pain)
Headache	–0.02	0.70
Pain in jaw	0.41	0.34
Pain in neck	0.10	0.52
Pain in forehead	–0.05	0.77
Difficulty when opening mouth	0.72	–0.01
Noise when opening closing mouth	0.72	–0.03
Difficulty while eating	0.60	0.02

### Gender Comparisons

Females had significantly higher TMD-7 scale scores and higher ratings for headache ( *p* < 0.001), pain in jaw ( *p* = 0.001), pain in neck ( *p* < 0.001), pain in forehead ( *p* = 0.001), difficulty opening mouth ( *p* = 0.011), and difficulty when eating ( *p* = 0.010) ( Table 6 ).

**Table 6:** Prevalence of TMD-7 items by gender

Item	Gender	Rarely or never	A few times per month	Once or twice a week	Nearly every day	*p* -Value
Headache	Male	60 (60%)	25 (25%)	12 (12%)	3 (3%)	< 0.001
	Female	61 (26%)	101 (44%)	56 (24%)	14 (6%)	
Pain in jaw	Male	79 (79%)	18 (18%)	2 (2%)	1 (1%)	0.001
	Female	145 (63%)	51 (22%)	26 (11%)	10 (4%)	
Pain in neck	Male	51 (51%)	31 (31%)	12 (12%)	6 (6%)	< 0.001
	Female	66 (28%)	75 (32%)	47 (20%)	44 (19%)	
Pain in forehead	Male	74 (74%)	19 (19%)	6 (6%)	1 (1%)	< 0.001
	Female	115 (50%)	73 (31%)	35 (15%)	9 (4%)	
Difficulty when opening mouth	Male	95 (95%)	3 (3%)	0 (0%)	2 (2%)	0.011
	Female	189 (81%)	28 (12%)	10 (4%)	5 (2%)	
Noise when opening closing mouth	Male	79 (79%)	13 (13%)	3 (3%)	5 (5%)	0.053
	Female	166 (72%)	29 (13%)	10 (4%)	27 (12%)	
Difficulty while eating	Male	94 (94%)	4 (4%)	0 (0%)	2 (2%)	0.010
	Female	189 (81%)	22 (9%)	16 (7%)	5 (2%)	

### Age Comparisons

No statistically significant differences were found in the TMD-7 scale score or the individual TMD-7 item ratings between age categories ranging from age groups 18 to 35, 36 to 50, and greater than 50 years of age ( *p* = 0.993) ( Table 7 ).

**Table 7:** Prevalence of TMD-7 items by age groups

Item	Age (y)	Rarely or never	A few times per month	Once or twice a week	Nearly every day	*p* -Value
Headache	18–35	24 (40%)	22 (37%)	13 (22%)	1 (2%)	0.50
	36–50	67 (32%)	88 (42%)	42 (20%)	11 (5%)	
	> 50	30 (47%)	16 (25%)	13 (20%)	5 (8%)	
Pain in jaw	18–35	39 (65%)	12 (20%)	8 (13%)	1 (2%)	0.39
	36–50	143 (69%)	45 (22%)	15 (7%)	5 (2%)	
	> 50	42 (66%)	12 (19%)	5 (8%)	5 (8%)	
Pain in neck	18–35	22 (37%)	20 (33%)	6 (10%)	12 (20%)	0.92
	36–50	71 (34%)	67 (32%)	40 (19%)	30 (14%)	
	> 50	24 (38%)	19 (30%)	13 (20%)	8 (13%)	
Pain in forehead	18–35	36 (60%)	14 (23%)	8 (13%)	2 (3%)	0.99
	36–50	116 (56%)	62 (30%)	24 (12%)	6 (3%)	
	> 50	37 (58%)	16 (25%)	9 (14%)	2 (3%)	
Difficulty when opening mouth	18–35	53 (88%)	5 (8%)	1 (2%)	1 (2%)	0.72
	36–50	177 (85%)	18 (9%)	8 (4%)	5 (2%)	
	> 50	54 (84%)	8 (13%)	1 (2%)	1 (2%)	
Noise when opening closing mouth	18–35	48 (80%)	6 (10%)	2 (3%)	4 (7%)	0.32
	36–50	155 (75%)	24 (12%)	9 (4%)	20 (10%)	
	> 50	42 (66%)	12 (19%)	2 (3%)	8 (13%)	
Difficulty while eating	18–35	48 (80%)	8 (13%)	4 (7%)	0 (0%)	0.60
	36–50	183 (88%)	11 (5%)	9 (4%)	5 (2%)	
	> 50	52 (81%)	7 (11%)	3 (5%)	2 (3%)	

### Orthodontic Treatment Comparisons

No statistically significant differences were found in the TMD-7 scale score or the individual TMD-7 item ratings between subjects with and without previous orthodontic treatment ( *p* = 0.075) ( Table 8 ).

**Table 8:** Prevalence of TMD-7 items by previous orthodontic treatment history

Item	Previous orthodontic treatment	Rarely or never	A few times per month	Once or twice a week	Nearly every day	*p* -Value
Headache	Yes	37 (29%)	51 (40%)	32 (25%)	6 (5%)	0.068
	No	84 (41%)	75 (36%)	36 (17%)	11 (5%)	
Pain in jaw	Yes	77 (61%)	33 (26%)	11 (9%)	5 (4%)	0.15
	No	147 (71%)	36 (17%)	17 (8%)	6 (3%)	
Pain in neck	Yes	41 (33%)	42 (33%)	21 (17%)	22 (17%)	0.39
	No	76 (37%)	64 (31%)	38 (18%)	28 (14%)	
Pain in forehead	Yes	70 (56%)	35 (28%)	16 (13%)	5 (4%)	0.53
	No	119 (58%)	57 (28%)	25 (12%)	5 (2%)	
Difficulty when opening mouth	Yes	108 (86%)	13 (10%)	1 (1%)	4 (3%)	0.95
	No	176 (85%)	18 (9%)	9 (4%)	3 (1%)	
Noise when opening closing mouth	Yes	95 (75%)	12 (10%)	5 (4%)	14 (11%)	0.84
	No	150 (73%)	30 (15%)	8 (4%)	18 (9%)	
Difficulty while eating	Yes	108 (86%)	5 (4%)	9 (7%)	4 (3%)	0.37
	No	175 (85%)	21 (10%)	7 (3%)	3 (1%)	

## Discussion

The main finding of this study was that the TMD-7 had an acceptable internal consistency. With a Cronbach's *α* of 0.77, this value indicates there is a correlation among the individual items or symptoms listed in the TMD-7.

It was predicted that the TMD-7 should be a 2-factor or subscale survey identifying between pain and function subscales. The statistical analysis showed the seven items should be used in a single scale and not divided into two factors. In future uses with this survey, it is possible that when the sample includes a larger proportion of patients with TMD, there may be a clearer indication of two factors within patients with TMD. If there are two factors within the TMD patients, the TMD-7 scale may have two uses: (1) use the single scale for identifying TMD patients and (2) use the two subscales for identifying different aspects of TMD within the TMD patients.

All items in the TMD-7 were correlated with a moderately positive correlation of 0.59 for the item's headache and pain in forehead and for the item's difficulty opening mouth and noise when opening and closing. 14 These make sense especially for headache and pain in forehead due to the association people make with a headache being associated with the temple and forehead regions. However, none of the seven items were strongly correlated due to the wide array of how TMD symptoms can present in a patient.

The results of the study demonstrated that females had significantly higher TMD-7 scale scores than males. With females having statistically significant higher ratings for headache, pain in jaw, pain in neck, pain in forehead, difficulty opening mouth, and difficulty when eating. These results align with current literature. A retrospective study by Bagis et al evaluated the prevalence of TMD symptoms of patients suffering from TMD and concluded females had a higher prevalence of TMD symptoms. 15 They reported TMJ pain at rest and masseter muscle pain being the most significant symptoms reported, with pain being the most common problem. 15 In a more recent longitudinal study by Häggman-Henrikson et al, they found that the prevalence of orofacial pain was reported higher than man (odds ratio 2.58, 95% CI). 16 In a systematic review and meta-analysis, Bueno et al concluded the odds of presenting with TMD were 2.2 times higher in women than men. 17 Moreover, pain and other nonpain symptoms have consistently been shown to be more prevalent in women. It is plausible that gender predominance exists due to biological and psychological characteristics that differ. This could be attributed to hormonal imbalances or even women's perception of pain. 18 19 20 21

The results of this study found no significant difference in TMD symptoms among age groups. Within the literature there was some conflicting data compared with our results. Bagis et al found age had significant effects on the prevalence of TMD as we age. 15 This does not support initial claims of TMD symptoms starting in an adolescent population, but due to comorbidities, hormone changes, and inflammatory disease that may develop with age, can contribute to the development of TMD in some individuals. 1 3 6 13

Our results indicated there was not a statistical difference in the TMD symptoms between orthodontically treated patients and those that had not completed orthodontic treatment. In a longitudinal cohort study of patients and controls, Hirata et al found no difference in the incidence of TMD signs and symptoms between treated and untreated subjects. 22 Conti et al conducted a cross-sectional study comparing signs and symptoms of TMD and orthodontic treatment demonstrated orthodontics does not predispose patients to TMD, with 62.5% of sample were considered TMD symptom-free. 23 With previous orthodontic treatment being implicated as a cause of TMD, more and more evidence seems to deny this claim. Magnusson et al's 20-year longitudinal study concluded that orthodontic treatment did not run a higher risk of developing TMD later in life. 24 Our results align with current literature for prior orthodontic treatment not being linked to TMD. Further studies and higher quality evidence would be beneficial to support these results further.

## Limitations and Directions for Future Research

Our study has a couple important limitations. First, because participants did not undergo an independent criterion examination for TMD, we could not determine the diagnostic operating characteristics (sensitivity, specificity) nor optimal cutpoints in screening for TMD. Second, this was a convenience sample of individuals attending an orthodontics clinic, and therefore generalizability to other populations needs to be determined.

Our findings point to several directions for future research. Further utilization of the TMD-7 in a TMD affected patient population, blinded comparison to a criterion standard TMD evaluation, 25 and dissemination to a larger patient population are imperative to reach full validation of the survey. Also, assessing construct validity using a global measure of symptom status and other pain-related domains, 26 as well as examining convergent validity with other brief TMD measures would be desirable. 27 It is our hope that through this process, the TMD-7 will have two uses: to identify a patient suffering from TMD and to differentiate their symptoms through the pain and function subscales.

## Conclusion

In conclusion, the newly developed TMD-7 has good internal consistency and can be used reliably for assessment of TMD symptoms in adults. The pilot use of this tool revealed no significant differences between age groups or subjects with or without previous orthodontic treatment but did find a significant female gender predisposition for TMD symptoms.
